# The Role of Nuclear Antiviral Factors against Invading DNA Viruses: The Immediate Fate of Incoming Viral Genomes

**DOI:** 10.3390/v8100290

**Published:** 2016-10-22

**Authors:** Tetsuro Komatsu, Kyosuke Nagata, Harald Wodrich

**Affiliations:** 1 Microbiologie Fondamentale et Pathogénicité, MFP CNRS UMR 5234, Université de Bordeaux, Bordeaux 33076, France; tetsuro.komatsu@u-bordeaux.fr; 2 Department of Infection Biology, Faculty of Medicine, University of Tsukuba, Tsukuba 305-8575, Japan; knagata@md.tsukuba.ac.jp

**Keywords:** adenovirus, antiviral response, herpesvirus, incoming viral genomes, intrinsic immunity, IFI16, PML nuclear body

## Abstract

In recent years, it has been suggested that host cells exert intrinsic mechanisms to control nuclear replicating DNA viruses. This cellular response involves nuclear antiviral factors targeting incoming viral genomes. Herpes simplex virus-1 (HSV-1) is the best-studied model in this context, and it was shown that upon nuclear entry HSV-1 genomes are immediately targeted by components of promyelocytic leukemia nuclear bodies (PML-NBs) and the nuclear DNA sensor IFI16 (interferon gamma inducible protein 16). Based on HSV-1 studies, together with limited examples in other viral systems, these phenomena are widely believed to be a common cellular response to incoming viral genomes, although formal evidence for each virus is lacking. Indeed, recent studies suggest that the case may be different for adenovirus infection. Here we summarize the existing experimental evidence for the roles of nuclear antiviral factors against incoming viral genomes to better understand cellular responses on a virus-by-virus basis. We emphasize that cells seem to respond differently to different incoming viral genomes and discuss possible arguments for and against a unifying cellular mechanism targeting the incoming genomes of different virus families.

## 1. Introduction

Viruses are intracellular parasites and need to reach their site of replication in order to propagate; this requires overcoming several structural and molecular barriers of the host cell. For nuclear replicating DNA viruses, the final destination is the nucleus, but before nuclear import of the viral genome can take place they must pass several subcellular compartments. In addition, each compartment of the host cell is equipped with “sensors” and “effectors” against invading viruses to detect the invading virion for degradation immediately after infection or, if this fails, to target the viral genome to prevent viral gene expression. During virus entry, the first obstacles to overcome are the plasma and/or endosomal membranes. At the cell surface or in the lumen of the endosomal compartment, sensor molecules called toll-like receptors (TLRs) are expressed. These sensors recognize molecules characteristic of pathogens, including their DNA genomes (e.g., upon virus degradation in the lysosome) [1]. Pathogen recognition induces signaling pathways mediated by nuclear factor κ-light-chain-enhancer of activated B cells (NF-κB) and interferon (IFN) regulatory factor 3 (IRF3), leading to the production of pro-inflammatory cytokines and IFNs [1]. Although the expression of TLRs is mainly found in a subset of immune cells, additional sensors have been found in non-immune cells. Following the internalization or endosomal escape, viruses are released into the cytoplasm, where they can be exposed to a series of DNA sensors such as cyclic GMP-AMP synthase (cGAS) [2,3,4]. These sensor proteins are widely expressed and activate signaling pathways similar to TLRs [2,3,4]. However, in many cases of nuclear replicating DNA viruses, their genomes are protected by protein shells, i.e., capsids, during cytoplasmic transport and are released only immediately before nuclear import occurs. Consequently, these cytoplasmic DNA sensors have no or only a limited chance to contact viral genomes (e.g., when the capsids are degraded). Several lines of evidence, largely from herpes simplex virus-1 (HSV-1) studies (see Section 3), have suggested that nuclear antiviral factors also function as DNA sensors and effectors to target incoming viral genomes immediately upon nuclear entry [5]. This antiviral response by host nuclear factors is considered as a part of the “intrinsic immunity” [6,7] and may constitute a common mechanism against incoming genomes of nuclear replicating DNA viruses [5]. The idea of the “intrinsic immunity” or “restriction factors” was originally established during studies on retroviruses due to the fact that a subset of host proteins that are constitutively expressed can directly suppress viral infections [7,8].

The most prominent factors in nuclear antiviral responses, which have been implicated in targeting incoming viral genomes, are components of promyelocytic leukemia nuclear bodies (PML-NBs) and the nuclear DNA sensor IFI16 (interferon gamma inducible protein 16). PML-NBs, also called ND10, are distinct subnuclear domains, which can be observed as punctate dots in microscopy analyses [9,10]. PML-NBs are composed of nuclear factors that are Small Ubiquitin-like Modifier (SUMO)-modified as well as have SUMO-interacting motifs (SIMs), such as PML, Sp100, and death domain associated protein (Daxx) [9,10]. Therefore, protein interactions mediated by SUMO-SIM combinations were proposed to be a major mechanism for the PML-NB formation [9,10,11]. PML is the main protein of the bodies, and depletion of this factor results in loss of the subnuclear dot-like structures [6,9,12]. Sp100 is known to have several splicing isoforms [13] and functions as either transcriptional activator or repressor depending on the splicing patterns [6,14]. Daxx forms a chromatin-remodeling complex with the transcriptional regulator ATRX [15], which mediates deposition of a histone H3 variant H3.3 at specific genomic loci [16]. Daxx was also shown to associate with other epigenetic regulators, including HDACs (histone deacetylases) [17,18] and Dnmt1 (DNA methyltransferase 1) [19], indicating a repressive role in the regulation of chromatin structures [6]. The expression of PML-NB components, PML, Sp100 [20], and Daxx [21] (and thus the formation of the bodies) is known to be IFN-inducible [9], suggesting potential roles of (the components of) PML-NBs in pathogen-related processes. Although a detailed understanding how PML-NBs and/or its components antagonize viral propagation is missing, they appear, in part, to directly control viral gene expression programs through transcriptional and/or epigenetic repression activities described above. IFI16 was identified as a sensor that directly binds to foreign DNAs and stimulates the NF-κB and IRF3 signaling pathways [22]. In the study, it was also shown that nuclear translocation of NF-κB and IRF3 upon HSV-1 infection is inhibited by knockdown of IFI16 [22], indicating that this sensor protein can target viral DNA genomes in the infection context. Still, it remains unclear how and/or where IFI16 triggers the antiviral signaling [4]; while the original study showed translocation of the protein from the nucleus to the cytoplasm upon DNA transfection [22], other reports in viral systems suggested that IFI16 is recruited onto viral genomes within the nucleus [23,24] (see Section 3).

The sensing and detection of incoming viral DNA genomes by nuclear antiviral factors, particularly the involvement of PML-NBs, has been studied with a variety of viral systems, but to date HSV-1 remains the best-studied model in this topic [5]. Importantly, some (but not all) of the observations appear similar among different DNA viruses, leading to a generalized concept in which viral DNA genomes are immediately targeted by a set of nuclear antiviral factors in a common way [5,6,25]. By carefully reading the literature, however, we reach the conclusion that for many DNA viruses, the interplay between nuclear antiviral factors and incoming viral genomes immediately following genome import remains to be shown. Furthermore, our recent studies using adenovirus (Ad) go against the concept of direct genome detection by these nuclear factors, showing that freshly imported Ad genomes neither localize at PML-NBs nor are they targeted by its components or IFI16 upon nuclear entry [26,27]. In this review, we aim to provide a comprehensive description of nuclear antiviral mechanisms targeting viral genomes upon nuclear import in the immediate early phase of DNA virus infection, by listing existing experimental evidence and discussing conclusions that have been drawn from limited examples including our own. We focus on the roles of nuclear antiviral factors against incoming genomes of several nuclear replicating DNA viruses. While there is no doubt that in several cases these factors exert antiviral activities in the nucleus, we believe that this may not be the result of a unique antiviral response against incoming viral genomes and suggest considering the roles of nuclear antiviral factors in this respect on a case-by-case basis. For cytoplasmic sensing of viruses or their genomes, please refer to several excellent reviews elsewhere [1,2,3].

## 2. Experimental Strategies to Study Nuclear Antiviral Factors

Most strategies to analyze the relationship between nuclear antiviral factors and viral genomes rely on biochemical and microscopic techniques. Chromatin immunoprecipitation (ChIP) assays can quantitatively detect how much a given (cellular or viral) factor is bound to specific genomic regions providing a single nucleotide resolution when combined with next generation sequencing (ChIP-seq) [28]. Although this biochemical assay is a powerful, presently widely used tool, the results generally reflect an average of cell populations and are “snapshots” of cells at a certain time, offering limited spatio-temporal resolution.

In contrast, microscopic, imaging-based analyses offer high spatial information (i.e., where a process takes place) at a single cell level. Localization of cellular and viral proteins can be visualized using specific antibodies in immunofluorescence (IF) analyses or through tagging with fluorescent proteins such as enhanced green fluorescent protein (EGFP) and mCherry. Visualization of viral genomes is technically more challenging. Fluorescence in situ hybridization (FISH) is the most straightforward way to detect specific genomes of interest; but requires harsh denaturing conditions, which may risk the integrity of subcellular structures. To overcome this problem, genome labeling with nucleoside analogs has been used as an alternative method. BrdU (5-bromo-2’-deoxyuridine) is a classical analog widely used for DNA labeling, but requires stringent denaturing for detection using antibodies, similar to FISH. However, when combined with nuclease treatments, incorporated BrdU can be detected with antibodies without denaturing, and this method has been used to visualize viral genomes such as Ad DNAs [26].

Furthermore, a novel genome labeling technique has recently been developed using “clickable” nucleoside analogs such as EdU (5-ethynyl-2’-deoxyuridine) and EdC (5-ethynyl-2’-deoxycytidine) [29]. These nucleoside analogs were shown to be substrates for viral DNA replication in a set of viral systems [29]. The advantage of this system is that viral genomes containing the analogs can be easily detected and visualized through click chemistry under mild sample treatment conditions that are compatible with IF analyses. Using this approach, Greber and co-workers succeeded to produce progeny Ad virions containing labeled genomes and to visualize single genomes of incoming viruses in early phases of infection [29]. This technique has also been used for visualizing incoming genomes of other viruses such as HSV-1 and papillomavirus [30,31,32]. To produce genome-labeled viruses, one may need to test several analogs, since labeling efficiency as well as cellular/viral toxicity varies among them. Indeed, it has been shown that high concentrations of certain analogs inhibit viral propagation [29], giving rise to the possibility that chemical modifications of viral genomes may change chemical/physical properties of the labeled DNA. Alternatively, viral and/or cellular factors that specifically bind to viral genomes can be used as a surrogate marker for genome localization (e.g., infected cell polypeptide (ICP) 4 of HSV-1, protein VII or template activating factor-I (TAF-I) in the case of Ad, see Section 3 and Section 5). Use of such protein markers, in combination with fluorescent proteins, allows simultaneous observation of viral genomes and nuclear antiviral factors in living cells (live-cell imaging), which provides a much higher spatio-temporal resolution over fixed IF samples. As targeting of nuclear antiviral factors against viral genomes can take place within minutes [5], development of live-cell imaging setup has greatly helped to understand cellular responses upon viral infection. On the downside, these imaging analyses sometimes result in high cell-to-cell variations and require many replicates and credible positive/negative controls in the experimental setup. Thus microscopic results need to be interpreted carefully. In summary, the combination of biochemical and imaging approaches remains the best approach for a comprehensive analysis of the interplay between incoming viral genomes and the host cell.

## 3. Herpes Simplex Virus-1

Two decades ago, the first observations on the fate of incoming viral genomes were published by Ishov and Maul, who showed that PML-NBs are sites for deposition of incoming viral genomes and/or viral DNA replication [33]. As similar phenomena were observed with Ad, HSV-1, and simian virus 40 (SV40) in this study [33] and with human cytomegalovirus (HCMV) in a subsequent study [34], it was suggested that PML-NBs are general sites for genome localization of nuclear replicating DNA viruses. Following the original study, using HSV-1 the group of Everett extensively investigated the mechanisms of how incoming viral genomes interact with PML-NBs upon infection, making this virus the best-studied model. Initially the group found that ICP4, a viral transcription regulator encoded in the immediate early gene, forms nuclear foci that are co-localized with or juxtaposed to PML-NBs during immediate early phases of infection [35]. It was also shown that ICP4 does not form foci when expressed alone, and that ICP4 foci develop into DNA replication compartments in later phases of infection [35,36], suggesting that ICP4 foci might contain incoming viral genomes. This idea was subsequently demonstrated by immuno-FISH experiments showing co-localization between viral genomes and ICP4 in early phases of infection [37], validating ICP4 as a marker for incoming HSV-1 genomes.

Furthermore, Everett et al. developed a series of recombinant viruses expressing fluorescent protein-tagged ICP4 for live-cell imaging analyses, and demonstrated that PML-NB components are dynamically recruited onto viral genomes to form de novo PML-NB-like structures [35,37]. Importantly, although the recruitment of PML-NB components is observed with wild type HSV-1 infection, it can only be observed within a short time window [37], since ICP0, another viral immediate early gene product, degrades PML through its E3 ligase activity and thus leads to loss of PML-NBs [38]. Accordingly, ICP0-null mutant viruses, which exhibit strong growth defects, show more stable association with PML-NBs. Intriguingly, in the absence of ICP0, the association between PML-NB components and ICP4 foci appears stable and remains observed even after ICP4 foci develop into DNA replication compartments [36], suggesting that the components may also be involved in viral DNA replication. To clarify the roles of (the recruitment of) PML-NB components, Everett and co-workers conducted a series of knockdown experiments against several components (i.e., PML, Sp100, Daxx, and ATRX) and reported that depletion of each of the components partially rescues the growth defect of ICP0-null viruses but has no effect on viral propagation of wild type HSV-1 [39,40,41]. These findings are consistent with an earlier report using mouse cells that no major difference between PML^+/+^ and PML^−/−^ cells is observed during wild type HSV-1 infection [42]. However, a study of another group reported that knockdown of PML enhances wild type HSV-1 propagation [43]. Nonetheless, this series of depletion experiments identified PML-NB components as restriction factors for HSV-1 that can be efficiently counteracted by ICP0. The knockdown experiments also revealed that the absence of one PML-NB component does not affect the recruitment of other components onto HSV-1 genomes [39,40,41], suggesting that each component is independently translocated onto viral genomes.

Consistent with this, it was shown that simultaneous depletion of PML and Sp100 enhances ICP0-null HSV-1 propagation greater than single depletion of each factor [40]. Furthermore, mutational analyses indicated that the SIM of each PML-NB component is necessary for its recruitment onto HSV-1 genomes [44]. In contrast, it remains unclear what initial molecular event triggers the SUMO-SIM-dependent relocalization of PML-NB components upon HSV-1 infection. Nevertheless, these studies clearly elucidate a model in which incoming HSV-1 genomes activate an unknown antiviral mechanism(s) that induces relocalization of PML-NB components onto viral genomes in a SUMO-SIM-dependent manner, while ICP0 antagonizes this cellular response, in part, by disrupting the bodies. This idea, consolidated through HSV-1 studies, is the basis of the current model for the nuclear antiviral response against incoming viral genomes [5,6,25].

The initial study identifying IFI16 suggested that this nuclear DNA sensor could act against HSV-1 infection [22]. Rapid recruitment of IFI16 onto incoming HSV-1 genomes has been demonstrated by IF and live-cell imaging analyses [23,24]. The effect of IFI16 depletion on HSV-1 infection, however, remains somewhat controversial. Some studies reported that IFI16 knockdown enhances viral propagation of wild type HSV-1 [45,46], while others showed this enhancing effect only with ICP0-null mutants but not with the wild type [23,47]. In subsequent studies, ICP0 was initially suggested to degrade IFI16 in a proteasome-dependent manner [48]. However, it was also reported that the degradation occurs during HSV-1 infection even in the absence of ICP0 [23,49], and that ICP0 is likely to inhibit IFI16 recruitment onto viral genomes [23]. Most recently, it was suggested that IFI16 is differentially regulated during HSV-1 infection in a cell type-dependent manner [50], which may explain the controversial observations on its depletion effects as well as the role of ICP0. Importantly, the events involving IFI16 are likely to take place in the nucleus. Therefore, the mechanism of how IFI16 as “nuclear” DNA sensor can initiate “cytoplasmic” signaling pathways such as NF-κB and/or IRF3 signaling shown in the earlier study [22] remains unclear [4]. Cristea et al. showed that IFI16 can be acetylated within the NLS (nuclear localization signal), leading to cytoplasmic translocation [51]. Knipe and co-workers have recently reported that cGAS, a cytoplasmic DNA sensor, can interact with IFI16 in the nucleus and stabilize it, which activates the IRF3 signaling pathway through an unknown mechanism [52]. In addition, two independent studies have suggested that IFI16 may introduce repressive chromatin structures onto HSV-1 genomes [46,47], although it remains to be shown if this is an active process by the protein or merely a consequence of the NF-κB and/or IRF3 signaling-mediated repression. Intriguingly, it has been reported that knockdown of IFI16 reduces the efficiency of the recruitment of PML-NB components onto incoming HSV-1 genomes [23]. However, the absence of the recruitment was observed with only a limited population of knockdown cells [23], and the underlying mechanism is not known. In conclusion, a series of studies have highlighted an antiviral role of IFI16 against incoming HSV-1 genomes, as has been observed with PML-NB components.

## 4. Other Herpes Viruses

Similar to HSV-1, a member of the alpha-herpesvirus family, modulation of PML-NBs has been shown with the beta-herpesvirus family member HCMV during immediate early phases of infection. Following the original study using HSV-1, Ad, and SV40 [33], Ishov and Maul conducted a similar investigation into HCMV and found that both IE1 and IE2, viral immediate early gene products, localize at or next to PML-NBs [34]. Subsequently, it was reported that some nuclear IE2 foci contain HCMV genomes (confirmed by immuno-FISH analyses) and develop into DNA replication compartments in later phases of infection [53]. These observations indicated that similar to ICP4 of HSV-1, IE2 could bind to incoming HCMV genomes and be a marker for incoming viral DNA, suggesting that incoming HCMV genomes might target or be targeted by PML-NBs. Despite the similarities with the HSV-1 system, there are also differences. Unlike ICP4 [35], IE2 was also shown to form nuclear foci and localize at PML-NBs even in the absence of viral genomes [53], making it less clear whether PML-NB localization of incoming HCMV genomes is an intrinsic property of the incoming viral genome or dependent on IE2. In addition, the earlier work by Ishov and Maul showed, using immuno-FISH, that only a small portion of incoming HCMV genomes localize at PML-NBs at two hours post-infection (hpi) [34]. It should be noted, however, that the localization patterns of IE2 are different between IE2-transfected and HCMV-infected cells: i.e., the formation of nuclear IE2 foci is dependent on the presence of PML or intact PML-NBs in transfected cells, while in infected cells IE2 forms nuclear dots depending on viral genomes even in the absence of PML and is dynamically associated with PML-NBs, followed by dissociation from and dispersal of the bodies [53,54]. Thus, although possible, unless more direct evidence is provided, one should not consider that both HSV-1 and HCMV genomes interact with PML-NBs in the same way during immediate early phases of infection.

The study of Ishov and Maul demonstrated that IE1 alone is able to and sufficient to disperse PML-NBs, somewhat similar to ICP0 of HSV-1 [34]. In contrast, this phenotype induced by IE1 is probably mediated via the removal of SUMO modifications from, rather than degradation of, PML and Sp100 [6,55,56]. In addition to IE1, the tegument protein pp71 was shown to interact with Daxx [57] and to induce the proteasome-dependent degradation of Daxx [58] or disrupt the interaction between Daxx and ATRX [59]. Intriguingly, tegument proteins of gamma-herpesviruses, such as the open reading frame (ORF)75 of Kaposi’s sarcoma-associated herpesvirus (KSHV) [60] and BNRF1 of Epstein-Barr virus (EBV) [61], also have been shown to inhibit ATRX. Targeting of PML-NB components has also been observed with other herpesviral proteins [6,62], suggesting that similar or analogous mechanisms antagonizing PML-NB components may be widely shared among herpesviruses. The similarity of the mechanisms is further demonstrated by reports showing that the replication defect phenotype of the ICP0-null HSV-1 mutant could be rescued by the combination of HCMV pp71 and IE1 [56] as well as BNRF1 of EBV [63]. Similar to the work from the Everett group for HSV-1, Stamminger et al. carried out a set of knockdown experiments against PML, Daxx, and Sp100, and found that single knockdown of individual components promotes the propagation of (wild type) HCMV, but double knockdown of each combination exhibits further enhancement of this phenotype [64,65]. This confirms that PML-NB components are restriction factors that individually function against HCMV.

The involvement of IFI16 in early infection has also been investigated for several herpesviruses. For instance, knockdown of IFI16 has been shown to greatly enhance HCMV propagation [45], and ChIP assays have revealed the binding of this host factor to HCMV genomes [66]. In contrast, although microscopic analyses have been carried out in some studies [67], limited resolution of the provided images holds back the understanding of the spatio-temporal processes involving IFI16 for other herpesviral infections. Therefore, a simple but important question remains unanswered; do herpesviral genomes other than HSV-1 also encounter IFI16 immediately upon nuclear entry?

## 5. Adenovirus

Incoming Ad genome is one of the viral genomes found at PML-NBs in the initial study of Ishov and Maul [33]. In that report, however, the genome localization at PML-NBs was shown only at 4 hpi, but not at 1.5 hpi [33]. In addition, only 50% of incoming Ad genomes showed co-localization at 4 hpi in the study [33]. These observations are apparently distinct from those of HSV-1 as well as the concept that incoming viral genomes are immediately targeted by PML-NBs upon infection. Nevertheless, a detailed analysis of the localization of incoming Ad genomes has not been carried out until recently, in part, due to the lack of experimental tools. One specific feature of Ad genomes is that they form a chromatin-like structure with viral basic core proteins in the virion [68]. Protein VII is the major histone-like protein and remains associated with Ad genomes at least during the first hours of infection, as demonstrated by several biochemical and microscopic studies [69,70,71]. Thus, protein VII can be used as a surrogate marker for incoming Ad genomes in IF analyses [72]. This idea is further supported by the work of Greber, showing that the majority of incoming Ad genomes labeled with “clickable” analogs are protein VII-positive [29]. Furthermore, it has been reported that a host nuclear protein TAF-I/SET (SE translocation) is recruited onto incoming Ad genomes through the interaction with genome-bound protein VII, as demonstrated by IF and ChIP assays [69,70]. Based on this property, we recently developed a live-cell imaging technique, showing that EGFP-tagged TAF-I forms infection-specific nuclear puncta reflecting Ad genome localization in living cells [72]. Using these imaging tools, we have reported that incoming Ad genomes neither target nor are targeted by PML-NBs immediately after infection [26]. Furthermore, absence of co-localization between incoming Ad genomes and PML-NBs is neither cell type-specific nor reversed by IFN treatments, as has been shown by our and recent independent studies [26,27,73]. These findings would be the first experimental evidence that incoming genomes of nuclear replicating DNA viruses are not necessarily trapped by PML-NBs upon nuclear entry, though it remains possible that these observations are related to specific experimental conditions (see Section 7).

Even if incoming Ad genomes may not associate with PML-NBs upon infection, modulation of the bodies and inhibitory roles of its components during Ad infection is well established, in common with herpesviruses. The most prominent phenotype is a morphological change of PML-NBs induced by E4orf3, a viral early gene product. E4orf3 is shown to be necessary and sufficient to localize at and reorganize PML-NBs into track-like structures [74]. The group of Hearing reported that an IFN-induced antiviral response is mediated by PML and Daxx and is antagonized by E4orf3 [75]. Still, in general, changes induced by E4orf3 are not immediate early events and take place at a later time when viral DNA replication begins [76]. E1A, an Ad immediate early gene product, was also shown to localize at PML-NBs [74]. However, incoming genomes of non-replicative, E1-deleted Ad vectors also do not localize at PML-NBs [26], making it unlikely that the expression of immediate early genes such as E1A prevents genome targeting by the bodies. In contrast, protein VI and IX, minor capsid proteins also present in incoming virions, have been reported to interact with PML-NBs and/or its components [77,78], which may affect the interplay between incoming Ad genomes and the bodies (discussed in Section 7). Unlike in our studies [26,27], the original study of Ishov and Maul showed that a population of Ad genomes localize at PML-NBs at 4 hpi [33]. Because Ishov and Maul had also suggested PML-NBs as Ad DNA replication compartments [33], we focused on viral DNA replication factors and found that DBP, a viral single-strand DNA binding protein essential for DNA replication, can target PML-NBs in the absence of any other viral factors, including viral genomes [26]. Based on these findings, we suggested that incoming Ad genomes do not interact with PML-NBs per se, but upon DNA replication viral genomes recruit or are recruited into or juxtaposed to the bodies via DBP [26]. Furthermore, a recent report showed that SUMO2/3 is recruited into Ad DNA replication centers in late phases of infection [79]. Thus, in the case of Ad infection, PML-NB components and/or SUMO-dependent pathways might be involved in viral DNA replication rather than targeting incoming viral genomes, although the underlying molecular mechanisms are not clear. Dobner and co-workers have extensively performed knockdown experiments for PML-NB components in the Ad infection context [80,81,82,83]. Interestingly, in these studies, knockdown of Daxx, ATRX, Sp100, but not PML, promoted Ad propagation [80,81,82,83]. The same group reported that PML isoform II (PML-II) could function as a positive regulators for Ad infection [83]. However, a recent study by Leppard et al. revealed an opposite result, showing that Ad propagation is promoted by specific knockdown of PML-II [84]. Therefore, further studies are needed to clarify the role(s) of PML-II during Ad infection.

Several independent studies under IFI16 knockdown conditions have shown that depletion of IFI16 has no impact on Ad propagation [45,47]. This is yet another obvious difference to herpesviruses [45]. Despite not affecting Ad propagation, the studies left open the question of whether this nuclear DNA sensor can still target incoming Ad genomes or not. In addition, similar to PML-NB components and IFI16, a host protein PHF13/SPOC1 (survival-time associated PHD finger protein in ovarian cancer 1/PHF13) has been recently suggested as a novel restriction factor targeting incoming Ad genomes [85], without any support of microscopic evidence. Our recent study using direct imaging approaches for incoming Ad genome complexes showed that neither IFI16 nor PHF13/SPOC1 associates with incoming Ad genomes during immediate early phases of infection [27]. Our findings are thus in line with knockdown experiments for IFI16, but argue against the previously proposed antiviral model for PHF13/SPOC1. Taken together, based on imaging analysis there appear to be clear differences in the observed cellular response between Ad and herpesviruses, suggesting that incoming Ad genomes may not be subject to a variety of nuclear antiviral mechanisms that target herpesviruses (see Section 7).

## 6. Other DNA Viruses

The work of Ishov and Maul indicated that DNA replication of SV40, a member of polyomaviruses (PyVs), takes place at PML-NBs [33]. While employing immuno-FISH for genome detection, the initial study did not discriminate whether incoming viral genomes or replicating genomes are associated with the bodies. In the following study, the same group addressed this question by using transfection of plasmid DNAs containing several regions of SV40 genomes and found that the association with PML-NBs is likely to be dependent on the T antigen-mediated DNA replication [86]. These observations are somewhat similar to those of Ad infection, as PML-NB localization in both cases appears to be linked to viral DNA replication rather than immediate early infection events. A recent study on mouse PyV using detailed image analyses also found viral DNA replication taking place at PML-NBs [87]. Interestingly, in this study viral DNA replication compartments were even observed in PML-null cells without any morphological changes, and absence of PML was shown to have no effect on viral propagation [87]. In contrast to the other DNA viruses described above, modulation or morphological changes of PML-NBs have not been observed during both SV40 and mouse PyV infection [33,87]. An exception is BK virus infection, in which PML-NB reorganization, as well as relocalization of Daxx and Sp100 from the bodies, has been observed [88]. Again, changes in PML-NBs during BK virus infection were shown to be viral DNA replication-dependent [88]. In the case of BK virus, however, PML knockdown was also shown to have no effect on viral propagation [88]. Interestingly, BK virus infection can partially rescue ICP0-null HSV-1 infection, which suggests that reorganization of PML-NBs induced by this virus may have the potential to inhibit antiviral roles of the bodies [88]. Taken together, at current no study has directly addressed whether incoming PyV genomes associate with PML-NBs. In contrast, the existing data suggest that PML and/or PML-NBs might play neither a positive nor a negative role for PyVs, despite viral DNA replication taking place at the bodies. In addition, depletion of IFI16 has been shown not to affect the expression level of large T antigen in SV40-infected cells [47].

Papillomavirus is yet another interesting example of a nuclear replicating DNA virus that behaves differently from the other viruses studied to date. Day et al. reported, using BrdU-labeled pseudoviruses of bovine papillomavirus (BPV), that viral genomes as well as the minor capsid protein L2 localize at PML-NBs immediately after infection [89]. In this report, however, it was shown that viral gene expression is higher in PML-expressing cells than in PML-null cells, suggesting a positive role of PML in BPV infection [89]. A recent study on human papillomavirus (HPV) has identified SIM-like motifs in L2 and demonstrated that one of the SIM is critical for the binding of SUMO2 and the PML-NB targeting of L2 as well as viral genomes, contributing to efficient HPV infection [32]. These observations imply an active targeting of viral genomes and/or L2 towards PML-NBs. However, imaging analyses in recent studies using EdU-labeled viruses indicated that only a limited population of incoming viral genomes appear associated with PML-NBs [31,32]. When expressed alone in cells, HPV L2 was shown to bind to Daxx and lead to loss of Sp100 without affecting PML localization [90]. However, the functional role of L2-Daxx interaction remains to be determined, as a recent knockdown study showed no significant effect on HPV infection upon Daxx depletion [91]. In the same study, it was shown that Sp100 knockdown promotes viral transcription and thus viral propagation [91]. In summary, although Sp100 seems to exert an antiviral activity, an active targeting of papillomavirus genomes towards PML-NBs via L2 protein has been suggested, and surprisingly PML may act as a positive factor for viral propagation (see below). IFI16 was recently reported to be a negative regulator for HPV infection as demonstrated by knockdown experiments, although no co-localization between the protein and viral genomes has been observed [92].

## 7. Possible Mechanisms for Distinct Cellular Responses: Determinant and Consequence

As shown above, based on the currently existing experimental basis, there does not seem to be a general rule that applies to all different nuclear replicating DNA viruses in terms of the responses of nuclear antiviral factors against incoming genomes. We suggest at least two possible interpretations of the existing data. The first interpretation would assume that host cells have the potential to respond to different viruses using a common mechanism, but each virus has specific countermeasures that prevent genome targeting by nuclear antiviral factors to different degrees, explaining apparent differences in the cellular response. This view has been propagated to some extent in the literature [5,6,25]. An alternative interpretation is that the observed differences in a set of viral systems indeed reflect distinct cellular responses (or absence thereof) against each virus. The most prominent example for viral countermeasures against nuclear antiviral factors is ICP0 of HSV-1; wild type HSV-1 shows only transient interaction with PML-NBs and is less sensitive to knockdown of the PML-NB components, while the ICP0-null virus exhibits stable association with the bodies and is greatly rescued by depletion of PML-NB components [39,40,41]. Moreover, ICP0-null virus can also be partially rescued by overexpression of the pp71 and BNRF1 structural (but not structurally related) proteins from HCMV and EBV respectively [56,63]. Both proteins target and inactivate the Daxx-ATRX chromatin-remodeling complex, which is also a functional property of ICP0, suggesting that all three viruses have evolved antagonizing factors to control cellular repression mediated by Daxx-ATRX. This is also the case for KSHV, where ATRX depletion has no apparent effect on viral infection because of the efficient antagonism by ORF75 [60]. Intriguingly, BK virus—a member of PyVs—was also shown to partially rescue ICP0-null HSV-1 infection, suggesting that reorganization of PML-NBs induced by this virus may inhibit antiviral properties of the bodies [88]. Thus, although knockdown of PML has no effect on BK virus propagation itself [88], this may be due to an effect of an unidentified viral protein(s), which efficiently antagonizes PML and/or other PML-NB components. In the case of Ad, minor capsid proteins VI or IX have been reported to interact with PML-NBs [77,78]. Consequently, these proteins (or further uncharacterized proteins) may prevent PML-NB components from targeting incoming Ad genomes by antagonizing their function.

This (non-exhaustive) list of examples shows that many viruses appear to encode antagonists for PML-NB components in their structural proteins, which in some cases seem to be functionally interchangeable. Because they enter cells with the invading virion, they could in principle alter an immediate nuclear response against imported genomes. Still, the amount of antagonistic factors is limited by the amount of invading virions and probably one would expect them to act in a genome-specific way rather than on a global scale. This may be one of the reasons why antiviral effects of nuclear antiviral factors on incoming genomes, at least for herpesviruses, have been best studied at a low multiplicity of infection (MOI) [39,40,41,64,65]. In this respect, in our recent studies where we did not observe co-localization between incoming Ad genomes and PML-NBs, cells were infected only at a high MOI [26,27]. Under such experimental conditions, high amounts of input viral particles may provide sufficient antagonistic factors to efficiently antagonize or saturate cellular antiviral mechanisms. Therefore, it remains possible that the MOI employed could be the cause of the discrepancies between ours and earlier studies, although absence of co-localization between incoming Ad genomes and PML-NBs was found even in cells showing a small number of viral genomes in our studies [26,27]. In addition, although we and others reported no localization of incoming Ad genomes at PML-NBs using several cell types [26,73], specific cell models may exhibit distinct phenotypes. This is exemplified in a recent study showing a cell type-dependent regulation of IFI16 during HSV-1 infection [50]. Thus, MOI and cell type-specific actions of nuclear antiviral factors should be considered when investigating nuclear antiviral responses.

But what if host cells do not have a “master plan” against different DNA viruses, what could be the determinant(s) for the different responses of nuclear antiviral factors against incoming viral genomes? Currently, the clearest differences in immediate early antiviral responses have been observed between HSV-1 and Ad infections. There are several differences between HSV-1 and Ad, for instance, length of the genomes (approximately 150 and 36 kbp, respectively). More strikingly, Ad genomes remain associated with protein VII forming a chromatin-like structure even after nuclear entry, whereas HSV-1 genomes are likely unprotected at least immediately after nuclear import. Genome-bound protein VII has been reported to be beneficial for viral early gene expression in cells [71]. In addition, incoming HSV-1 genomes have been shown to recruit a set of cellular factors involved in the DNA damage response (DDR) upon nuclear entry [93], while Ad protein VII has been suggested to play a protective role against the DDR pathway [94]. However, the recruitment of DDR proteins onto HSV-1 genomes has been demonstrated to be a process independent from loading of PML-NB components [93]. Thus, another mechanism must exist to explain distinct responses of PML-NBs. Recent biochemical studies on IFI16 suggested a mechanism in which cellular nucleosomes inhibit the DNA recognition via the protein [95,96]. It was reported that IFI16 knockdown increases the SV40 early protein level (large T antigen) in cells transfected with a (naked) plasmid DNA encoding the viral genome, but not in the infection context where viral DNA is packed with histones in the virion [47]. Importantly, these experiments were performed under the same experimental conditions, using the same cell type (normal human foreskin fibroblasts) examined at the same time point (48 h post transfection/infection) [47], although it is generally difficult to directly compare plasmid transfection with infection experiments. These examples imply the importance of the chromatin state of a given viral genome upon nuclear entry in specific cellular antiviral responses. In this context, Everett et al. showed that IFI16 knockdown affects the recruitment of PML-NB components onto HSV-1 genomes [23]. Furthermore, a recent proteomic analysis on HSV-1 infected cells identified SUMO1 and PML as IFI16 interactors [49]. Thus, an attractive model is that IFI16 targets naked HSV-1 genomes, leading to the recruitment of PML-NB components in a SUMO-SIM-dependent manner, while genome-bound protein VII protects Ad genomes from the same fate. However, this model is certainly too simple because the effect of IFI16 knockdown was observed only with a small population of cells [23]. Intriguingly, Cristea and co-workers recently conducted an interactome analysis on the pyrin and HIN domain (PYHIN) family proteins, to which IFI16 belongs [43]. In the report, IFN-inducible protein X (IFIX), another PYHIN protein, was also shown to function as a nuclear DNA sensor, to interact with SUMO2 and PML-NB components, and to suppress HSV-1 infection [43]. For the scenario described above, this could mean that the PYHIN protein family could function redundantly or cooperatively upon HSV-1 infection (and other non-chromatinized viruses) to provide a link with PML-NBs, although either hypothesis remains to be tested.

Another possible mechanism for the interplay between viral genomes and nuclear antiviral factors is that viruses may actively interact with these host proteins via viral (genome-associated) proteins. A potential candidate for such a role is the L2 minor capsid protein of HPV, which was suggested to be responsible for the viral genome localization at PML-NBs [32,89]. HCMV IE2 could be another example for a viral protein, which on one hand is able to independently target PML-NBs, while on the other hand it was shown to co-localize with viral genomes at the bodies in the infection context, potentially linking the genome to PML-NBs [53]. In the case of Ad, DNA binding protein (DBP), a viral DNA replication factor, rather than incoming viral genomes, targets PML-NBs and thus may serve as recruiting factor for incoming viral genomes into the bodies during the onset of viral DNA replication [26], supporting the earlier observation that Ad DNA replication takes place at the bodies [33]. In support of this notion, SUMO proteins, critical regulators of PML-NBs, are also found in Ad and HCMV DNA replication compartments [79,97]. A potential link for viral DNA replication and PML-NBs was also reported for PyVs [33,87,88] and even for HSV-1 (in the absence of ICP0) [36], although in these cases a viral factor(s) responsible for the interplay between (incoming) genomes, replication compartment, and PML-NBs has not been identified. It is important to highlight that in some cases components of the PML-NBs might play a role in favor of viral genome regulations such as DNA replication, which would convert them into proviral factors. However, as indicated above, the knockdown of a given PML-NB component does not necessarily affect viral propagation in the same way when comparing different viral systems. As such the potential role of PML-NBs in viral processes, has to be seen as virus specific.

An interesting aspect of studying the immediate early fate of incoming viral genomes is whether nuclear antiviral factors are involved in the establishment and/or maintenance of latent infections. As observed, incoming Ad genomes are not encountered by nuclear antiviral factors and can efficiently initiate a lytic infection cycle, while incoming HSV-1 genomes (and possibly other herpesvirus genomes) are targeted by the restriction factors and frequently establish latency. Based on these observations, it is possible that the lytic-latent decision is made at the time of initial nuclear entry of viral genomes involving the activity of nuclear antiviral factors. It was shown in mice models that HSV-1 genomes locate at PML-NBs during acute phases of infection and during subsequent latent phases in neurons, and that absence of PML affects the expression of latency associated transcripts (LAT) [98]. In contrast, an early study on EBV failed to observe latent viral genomes at PML-NBs [99]. The group of Frappier showed that the viral nuclear protein EBNA1 (Epstein–Barr nuclear antigen 1) disrupts PML-NBs through degradation of PML during latent EBV infection and appears to contribute to viral reactivation [100,101]. An analogous mechanism has been observed with KSHV that K-Rta, a viral SUMO-targeting ubiquitin-ligase, promotes degradation of SUMO-modified proteins including PML and is essential for viral reactivation [102]. On the other hand, it was reported that several tested PML-NB components do not contribute to the establishment of KSHV latency [103]. Similarly, a HCMV study reported that knockdown of PML, Sp100, or Daxx is unlikely to affect the establishment of latent infection [104]. These data highlight again that, although PML-NBs and/or its components could be involved in the establishment and/or maintenance of herpesviral latency, it is unlikely that a common mechanism of interplay between PML-NBs and viral genomes during latency exists for all viruses. Intriguingly, a recent study on KSHV has suggested a role for IFI16 in maintenance of latent infection [105]. In summary, the potential role of known and yet to be discovered nuclear antiviral factors in latent infection is of major interest, and further studies are needed to understand how latency is established on incoming genomes.

## 8. Conclusion

In this review, we tried to focus the attention of the reader on the existing experimental evidence as well as the possible explanations of how nuclear antiviral factors target incoming DNA viruses immediately upon infection. While it remains possible that nuclear antiviral factors are able to target a variety of nuclear replicating DNA viruses in a similar fashion, we think that this should be experimentally validated in each viral system. The recent work done on HSV-1 and Ad has shown that significant differences in the cellular response exist; this could be explained by individually adapted nuclear responses of the host cell against each virus, rather than an overall antiviral strategy. In this respect, it is important to investigate on a virus-by-virus basis whether suspected immediate early events (e.g., interactions of nuclear antiviral factors with invading viral genomes) are indeed taking place and what could be the driving force(s) behind them. Novel techniques have been developed for viral genome detection that should pave the way to address these questions in greater detail. These techniques allow the visualization of single viral genomes of any virus of interest and thus may help to understand the underlying mechanisms of the confrontation between host cells and incoming viral genomes. In addition, if viral genomes are immediately sensed and repressed upon nuclear entry, viral (immediate) early gene expression should be affected by nuclear antiviral factors in early phases of infection. In many studies, however, depletion effects for cellular antiviral factors have been measured in later phases of infection (e.g., with progeny viral titers), thus potentially mixing early and late antiviral (or proviral) effects. Hence, we believe that a more direct investigation of the immediate early effects elicited by nuclear antiviral factors would help to describe the nuclear response to incoming genomes. Especially since the mechanistic details (and molecules) of initial genome detection remain poorly understood even for well characterized viral systems such as HSV-1 and Ad. Further detailed analyses using these and other viral models would lay the experimental foundation that could help to consolidate or revise the current view for the immediate early fate of incoming viral genomes.
